# Encapsulation of calcium carbonate with a ternary mixture of sodium caseinate/gelatin/xanthan gum to enhance the dispersion stability of solid/oil/water emulsions

**DOI:** 10.3389/fnut.2022.1090827

**Published:** 2022-12-12

**Authors:** Jie Zhang, Yanping Cao, Duoxia Xu

**Affiliations:** Beijing Key Laboratory of Flavor Chemistry, Beijing Laboratory for Food Quality and Safety, Beijing Advanced Innovation Center for Food Nutrition and Human Health (BTBU), Beijing Engineering and Technology Research Center of Food Additives, Beijing Higher Institution Engineering Research Center of Food Additives and Ingredients, School of Food and Health, Beijing Technology and Business University, Beijing, China

**Keywords:** calcium carbonate, sodium caseinate/gelatin/xanthan gum, stability mechanism, molecular docking, solid/oil/water emulsions

## Abstract

Calcium carbonate (CaCO_3_) has poor suspension stability, which severely limits its application in food processing and products. The solid/oil/water (S/O/W) emulsion stabilized by sodium caseinate (NaCas), gelatin (GEL), and xanthan gum (XG) ternary composite was to improve the dispersion stability of CaCO_3_ in emulsions. Particle size, Zeta potential, physical stability, and microstructure were determined to characteristic the stability of the S/O/W emulsions. Shear rheological and tribological analyses were used to characterize the rheological properties of S/O/W emulsions. X-ray diffraction (XRD), Infrared spectral analysis (FTIR), and molecular docking were used to characterize the molecular interactions, which was to explore the influence of the W phase on the system stability. It was found that when the NaCas concentration was 2 wt% and the S/O phase addition was 5%, the particle size distribution was uniform, and the physical stability was improved. CLSM and Cryo-SEM results showed that the S/O/W emulsions could embedded CaCO_3_ in the system, and formed a dense three-dimensional network space structure. The viscosity of the system increased and even agglomeration occurred with NaCas concentration increased, and the stability of the emulsion decreased. XRD results confirmed that the CaCO_3_ was partially covered due to physical embedding. Infrared spectral analysis and molecular docking results showed electrostatic, hydrophobic interaction, and hydrogen bond interaction between NaCas, GEL, and XG, which could improve the stability of S/O/W emulsions. The results showed that the S/O/W emulsions delivery system is an effective way to promote the application of CaCO_3_.

## Introduction

Calcium carbonate (CaCO_3_), with its high calcium content and low price, is commonly used in calcium fortification. Most calcium supplements on the market are tablets, which are not suitable for young children and people with swallowing difficulties. The emulsion system is convenient to swallow, but the insoluble calcium salt in the emulsion system is subject to the action of gravity and gravitational force (1), which is prone to precipitate during storage. CaCO_3_ is poor in suspension stability and easy to precipitate, which seriously limits its food processing and product application. In the field of food, milk powder is mainly supplemented with CaCO_3_, and the calcium supplement products on the market are mostly CaCO_3_. The main problem of dairy products with insoluble calcium salt is that insoluble calcium salt is easy to precipitate during storage. In the actual production process, micro-powdered calcium powder is usually mixed with dairy products. The researches were mainly included nano-CaCO_3_-sodium alginate-gelatin balls (2), nano-CaCO_3_ Pickering emulsion (3), and micro-powdered calcium powder, etc., which effectively improve the storage stability and calcium absorption rate. However, there is little research on traditional CaCO_3_ powder in food emulsions. In this study, the S/O/W emulsion delivery carrier was constructed to provide a reference for solving the dispersion problem of insoluble CaCO_3_ in liquid food. Studies have found that the delivery of solid-phase nutrients by S/O/W (Solid-in-Oil-in-Water) technology has become a new delivery carrier (4) with the advantages of low cost and simple preparation. Currently, the S/O/W technology was mainly focused on the embedding protection of proteins, enzymes, vitamins, probiotics, and other vulnerable nutrients (5–8). However, the application of mineral elements is rarely studied. The formation of CaCO_3_-loaded particles based on S/O/W emulsions is a promising method to improve the dispersion stability and bioavailability of CaCO_3_ in liquid foods.

Proteins and polysaccharides are essential in the production and stabilization of emulsions, mainly due to their ability to reduce the interfacial tension of droplets and improve the viscosity of continuous phases (9–11). Since there are both hydrophilic and hydrophobic groups in the protein structure, such macromolecules can be adsorbed at the interface of oil and water phase droplets, thus improving the stability of the emulsion. Sodium caseinate (NaCas) is a natural emulsifier with high nutritional value. In the emulsification process, NaCas can quickly adsorbs on the oil-water interface, reduces the interfacial tension, and forms a thick interfacial layer. Through electrostatic repulsion and steric hindrance, it can prevent the flocculation and condensation of the newly formed droplets, thus extending the stability of the droplets in the emulsion (12). Gelatin (GEL) contains both hydrophilic and hydrophobic groups and is used as an emulsifier (13). Polysaccharides are widely used as thickeners, stabilizers, emulsifiers, and gelling agents in the food industry. Nonadsorbing polysaccharides can not only increase the continuous phase viscosity and inhibit coalescence but also enhance the adsorption performance of proteins at the oil-water interface (14). Xanthan gum (XG) is widely used to improve the stability of emulsions because it can significantly increase the viscosity of the emulsion dispersed phase or form a gel network structure (15).

When GEL is used as a single emulsifier, the emulsions tend to form large droplets with poor stability (12, 16, 17), limiting their application in food processing. The binary blend of NaCas and GEL can change the original polymer spatial structure and better penetrate the NaCas network structure. Composite biofilms or microgels prepared using NaCas-GEL interactions have a wide range of applications in food packaging and biopharmaceuticals (18). In general, XG cannot form gels independently. NaCas stabilized emulsions have rapid phase separation at low concentrations (1.5∼4%), while with the addition of XG, both repulsive flocculation and continuous phase viscosity increase, and emulsion stability is improved (19). The interaction between NaCas and XG was closely related to the adsorption of NaCas at the oil-water interface, and the complex formed at acidic pH had a significant effect on the dynamic properties of the NaCas adsorption membrane (20). In addition, the adsorption rate of NaCas-XG mixtures decreased due to the high viscosity of XG and/or the formation of NaCas-XG complexes in the aqueous phase, and at higher protein concentrations, NaCas-XG interactions increased the surface swelling elasticity (21). NaCas and XG form weak non-covalent complexes through hydrophobic or electrostatic interactions, changing the nature of the bulk phase and the interfacial layer of emulsion droplets. However, the ternary mixture of NaCas, GEL (positively charged), and XG (negatively charged) in emulsions has not been reported.

In this study, S/O/W emulsions were constructed using CaCO_3_ as the S phase, soybean oil as the O phase, and the W phase containing NaCas-GEL-XG. The particle size distribution, Zeta-potential, physical stability, rheology, and microstructure of the emulsions prepared with different NaCas concentrations and different S/O phase additions were investigated to explore the formation pattern. The stabilization mechanism of S/O/W emulsions was analyzed by X-ray diffraction analysis, Infrared spectral analysis, and molecular docking techniques. This study lays the theoretical foundation for the design of three-phase (S, O, W) matrix delivery systems.

## Materials and methods

### Materials

Calcium carbonate was purchased from Shuang Teng Industrial Co., Ltd. (Henan, China). NaCas (Lot#C10185129) and XG were obtained and acquired from Macklin Biochemical Co., Ltd. (Shanghai, China). GEL was bought from Sinopharm Chemical Reagents Co., Ltd. (Beijing, China). Soybean Oil was provided by COFCO Co., Ltd. (Tianjin, China). Nile Blue A and Nile Red were procured from Sigma-Aldrich Co. (St. Louis, MO, USA). All other chemicals were analytical grade acquired from Sinopharm Group Co., Ltd (Beijing, China).

### Preparation of calcium carbonate solid/oil/water emulsions

NaCas (2.0 wt%, 4.0 wt%, 6.0 wt%, 8.0 wt%, 10.0 wt%), GEL (8.0 wt%), and XG (0.5 wt%) solutions were individually prepared using PBS buffer (1.0 mM, pH 7.0). These solutions were stirred in a water bath at 50°C for at least 2 h and then stored overnight at 4 °C to ensure adequate dissolution.

Calcium carbonate powder (S phase) and soybean oil (O phase) were accurately weighed with the ratio of 1:10, and stirred at 700 rpm for 1 h. Then the samples were stirred using a high-speed mixer for 3 min at 15,000 rpm (ULTRA TURRAX T25 Digital, IKA, Staufen, Germany) to form the S/O phase suspension.

Different concentrations of NaCas, GEL and XG solution were accurately weighed and mixed evenly at a ratio of 1:1:1 (*w/w/w*) (aqueous phase, 95%, 90%, 85%, 80%, 75%, W/w), and then a certain amount of S/O phase suspension (S/O phase, 5%, 10%, 15%, 20%, 25%, W/W) was added. The S/O/W emulsion was prepared by shearing at 15000 rpm for 5 min with a high-speed disperser (22).

### Particle size, Zeta-potential, and physical stability measurements

Particle size measurements were performed using a laser diffractometer (S3500 Microtrac Inc, Largo USA) with a refractive index of 1.51 and a dispersive medium refractive index of 1.33. The polydispersity index (PDI) based on the distribution of volume parts was calculated according to the following formula:


$$\text{PDI}=\frac{{\text{D}}_{90}-{\text{D}}_{10}}{{\text{D}}_{50}}$$

where D_10_, D_50_, and D_90_ were volume diameters equivalent to 10, 50, and 90% of the cumulative volume, respectively.

The Zeta-potential was measured by a Zetasizer Nano-ZS90 (Malvern Instruments, Worcestershire, UK). The electrophoretic mobility was measured in 11 consecutive readings after the sample was equilibrated for approximately 120 s.

Physical stability measurements were performed using a LUMiSizer (LUM GmbH, Berlin, Germany). The LUMiSizer stability analyzer can quickly determine the stability of emulsion by accelerating stratification, quantifying precipitation, and suspension. The experimental results are represented by the original transmission curve. The instability index can be obtained by further analysis and calculation of the original transmission curve through software, which can quantitatively represent the physical stability of the emulsion. The injection volume was 0.4 mL, rotation speed was 2000 rpm, time interval was 10 s, scanned 200 times, and test temperature was 25°C.

### Viscosity measurements

HAAKE rheometer (MARS IQ Air, HAKKE, Germany) was used to measure the apparent viscosity of the samples. When adding samples, pay attention to keeping the sample uniform to prevent bubbles. Select the plate rotor CC25 DIN, shear rate was 2∼200 S^–1^ at 25°C.

### Friction coefficient

The friction coefficient was performed by a rheometer (TA, DHR-1, America) to analyze. A full-ring stainless steel probe was used to simulate the surface of the mouth with polydimethylsiloxane (PDMS) to measure the lubrication properties of the samples. The preparation method of PDMS: mixed basic fluid and crosslinking agent with the ratio (10:1, w/w), prepared the PDMS with surface roughness (Ra) < 50 nm, vacuumized to remove bubbles and then cured at 70°C overnight.

The sample was placed on the ball-disk surface to simulate the friction process between the tongue surface and the roof of the mouth. As the device rotates, the system automatically records the tribological behavior of the sample. The parameters are as follows: test pressure 1 N, test temperature 37°C. Three independent replications were conducted for each sample.

### Microstructure analysis

#### Confocal laser scanning microscopy microstructure

Confocal scanning laser microscopy (CLSM) (FV3000, Olympus, Japan) was employed on the microstructure of CaCO_3_ S/O/W emulsions. Soybean oil (O phase) was pre-stained with Nile red (488 nm laser excitation source), NaCas and GEL were stained with Nile blue A (635 nm laser excitation source) before emulsification. The samples were magnified using a 10× eyepiece and a 60× objective lens (oil immersion) (23).

#### Cryo-scanning electron microscopy microstructure

Cryo-scanning electron microscopy (Helios NanoLab G3 UC, FEI, USA) was employed on the cross-sectional and interfacial structure of CaCO_3_ S/O/W emulsions. Samples were placed in liquid nitrogen and then transferred to a cryo-preparation chamber (PP3010T, Quorum Technologies, UK) under vacuum. After freeze-fracturing and high vacuum sublimation to sublimation at −95°C for 10 min to unbound water, and then the samples were sputter-coated with platinum and imaged using SEM. The observation was carried out at a distance between 3 and 5 mm with TLD detection at 2 kV (22).

### X-ray diffraction

An X-ray diffractometer (BRUCKER D8 ADVANCE, Brooke, Germany) was used to analyze the S/O/W emulsions. Samples were freeze-dried to get the powder. The spectral range was 5∼50 degrees, the scanning rate was set to 2 degrees/min, the acceleration voltage was 40 kV, and the tube current was 40 mA (24).

### Infrared spectral analysis

A FTIR Spectrometer (IS10, Thermo Nicolet Corporation, Waltham, MA, USA) was used to analyze the S/O/W emulsions. Samples were freeze-dried to get the powder. The wavenumber range was 400∼4000 cm^–1^, the resolution was 4 cm^–1^, the signal to emission ratio was 50000:1, and the spectrometer scanned 64 times (25).

### Molecular interconnection

Molecular docking technology was used to elucidate and determine the possible binding modes between different W phases. NaCas, GEL, and XG 3D structured PDB files were searched and collected in the https://pubchem.ncbi.nlm.nih.gov/ database. The software AutoDockTools-1.5.7 was used to perform the hydrogen addition, nucleation, and merging of non-polar hydrogen processing followed by a backup. Molecular docking was performed using Autodock Vina 1.5.7 to determine the mode of interaction of the receptor with the ligand using the affinity (kcal/mol) as an indicator.

### Statistical analysis

Each experiment was performed at least in triplicate. The results were presented as means ± standard deviations. Statistical analysis was performed using Origin 8.5 software and the SPSS 17.0 statistical analysis system (SPSS Inc., Chicago, IL, USA) according the one-way analysis of variance (ANOVA) method, and the significance level (P) was 0.05.

## Results and discussion

### Characteristics of calcium carbonate solid/oil/water emulsions

#### Particle size

**FIGURE 1:**
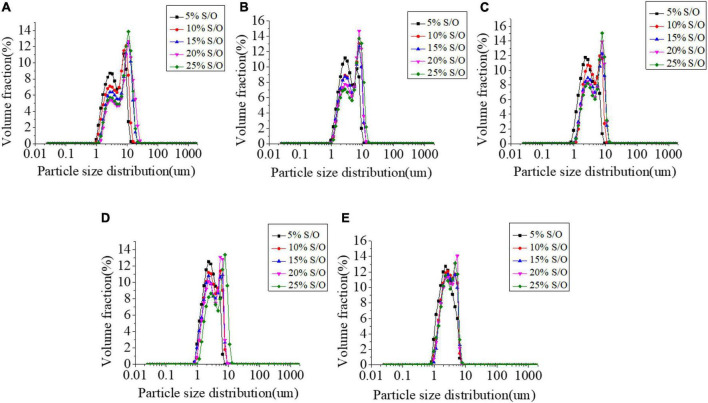
Effect of the different sodium caseinate (NaCas) concentrations **(A–E)** 2 wt%, 4 wt%, 6 wt%, 8 wt%, and 10 wt%) and different S/O phase addition on the particle size distribution of CaCO_3_ S/O/W emulsions.

The particle size distribution has a great influence on the stability of the emulsions system (26). The emulsion is a thermodynamically unstable system with two kinds of macroscopic instability, one is the migration of dispersed phase particles, and the other is the change of particle size of dispersed phase particles. Droplet size distribution is one of the most important characteristics of emulsions and is essential for system stability. In general, the emulsions are more stable with a narrower particle size distribution and smaller average particle size. The effects of different NaCas concentrations and S/O phase additions on the particle size of S/O/W emulsions were shown in Figure 1 and Table 1. The results show that when the S/O phase addition amount was constant, the NaCas-GEL-XG ternary composite stabilized S/O/W emulsion has a large distribution range and a relatively large average particle size with low NaCas concentration (2 wt%). In contrast, the S/O/W emulsions prepared by NaCas-GEL-XG as the W phase had a narrower particle size distribution range than prepared by NaCas, NaCas-GEL, and NaCas-XG, which was enhanced overall stability (22, 27). This was because the addition of GEL enhanced the emulsion stability performance of the system, and the addition of XG improved the viscosity and suspension stability of the system. With the NaCas concentration increased, the change of the particle size distribution was not obvious. When NaCas concentration was constant, the size distribution range became larger with the S/O phase addition increased, and the S/O/W emulsion system tended to be unstable.

**TABLE 1:** The average particle size for solid/oil/water (S/O/W) emulsions stabilized by different sodium caseinate (NaCas) concentrations (2∼10 wt%) and different S/O phase addition (5∼25%).

NaCas concentration (wt%)	5% S/O	10% S/O	15% S/O	20% S/O	25% S/O
2	3.74 ± 0.1^f^	4.18 ± 0.1^e^	4.31 ± 0.1^e^	5.36 ± 0.22^e^	5.8 ± 0.23^e^
4	2.89 ± 0.12^d^	3.05 ± 0.11^d^	3.23 ± 0.11^d^	3.77 ± 0.10^e^	4.52 ± 0.14^e^
6	1.81 ± 0.13^b^	1.89 ± 0.06^b^	1.97 ± 0.1^b^	2.1 ± 0.08^c^	2.41 ± 0.17^c^
8	1.46 ± 0.12^a^	1.67 ± 0.07^b^	1.86 ± 0.08^b^	1.84 ± 0.1^b^	2.11 ± 0.12^c^
10	1.23 ± 0.11^a^	1.64 ± 0.11^a^	1.68 ± 0.06^b^	1.69 ± 0.13^b^	1.94 ± 0.19^b^

Values for each samples with the same letter are not statistically different.

#### Zeta-potential

**FIGURE 2:**
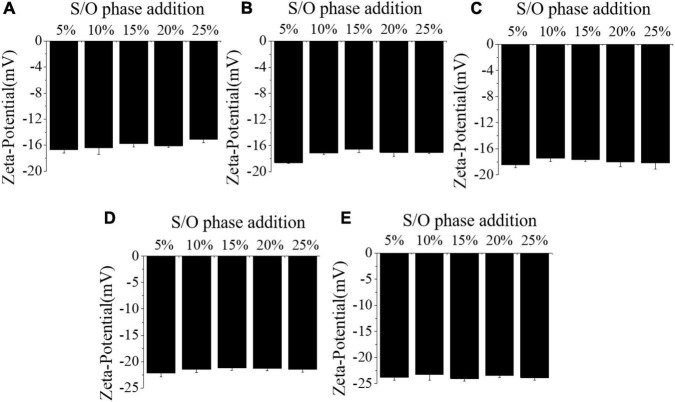
Effect of the different NaCas concentrations **(A–E)** 2 wt%, 4 wt%, 6 wt%, 8 wt%, and 10 wt%) and different S/O phase addition on the Zeta-potential of CaCO_3_ S/O/W emulsions.

The electrical properties of S/O/W emulsions are often characterized by Zeta-potential. The effects of different concentrations of NaCas and S/O phase addition amount on the Zeta-potential of S/O/W emulsions were shown in Figure 2 and Table 2. When the S/O phase added amount was constant, the absolute value of the Zeta-potential of microspheres was relatively small at low NaCas concentration, and slightly increases at high NaCas concentration. With the S/O phase addition increased, the absolute value of the microsphere potential decreased slightly and then increased. Compared with NaCas, NaCas-GEL, and NaCas-XG in phase W, the potential value of the microspheres was lower, which was due to electrostatic interaction in the mixed system. S/O/W emulsions (pH7.0) were negatively charged due to pH higher than the pI of NaCas, while GEL was positively charged, and electrostatic interaction between NaCas and GEL occurs, which was the result of interaction between GEL side groups (−NH^3+^) and negative ions dissociated from NaCas (28). The addition of XG increased the absolute value of Zeta-potential slightly due to the negative charge caused by the presence of anionic polysaccharide.

**TABLE 2:** The Zeta-potential for S/O/W emulsions stabilized by different NaCas concentrations (2∼10 wt%) and different S/O phase addition (5∼25%).

NaCas concentration (wt%)	5% S/O	10% S/O	15% S/O	20% S/O	25% S/O
2	−16.6 ± 0.57^c^	−16.4 ± 0.6^d^	−15.77 ± 0.57^d^	−15.9 ± 0.51^d^	−15.1 ± 0.29^d^
4	−18.6 ± 0.1^c^	−17.1 ± 0.56^c^	−16.57 ± 0.38^e^	−17.0 ± 0.21^c^	−17.0 ± 0.55^c^
6	−18.4 ± 0.32^c^	−17.46 ± 0.21^c^	−17.77 ± 0.11^c^	−18.0 ± 0.80^c^	−18.1 ± 0.92^c^
8	−22.2 ± 0.72^b^	−20.5 ± 0.98^b^	−19.17 ± 0.47^c^	−21.3 ± 0.43^b^	−21.5 ± 0.52^b^
10	−23.8 ± 0.51^a^	−23.27 ± 1.07^a^	−24.03 ± 0.64^a^	−23.4 ± 0.38^a^	−23.8 ± 0.63^a^

Values for each samples with the same letter are not statistically different.

#### Physical stability

The stability analyzer can be used to directly measure the physical stability of the S/O/W emulsions. When the instability index is lower, the emulsions maintain good physical stability.

**FIGURE 3:**
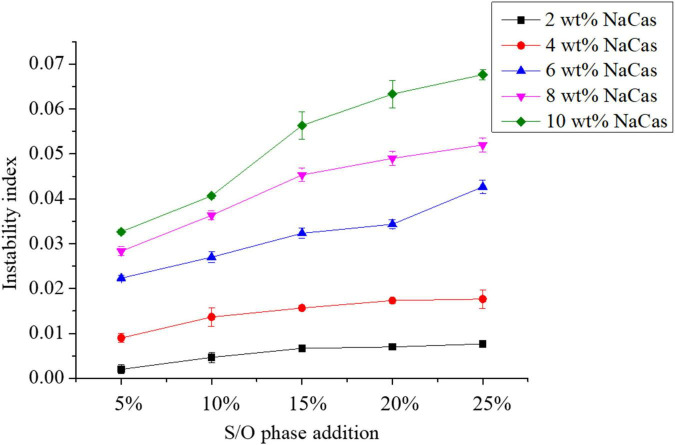
Effect of the different NaCas concentrations 2 wt%, 4 wt%, 6 wt%, 8 wt%, and 10 wt%) and different S/O phase addition on the physical stability of CaCO_3_ S/O/W emulsions.

The instability index of S/O/W emulsions prepared with different NaCas concentrations and S/O phase addition amounts was shown in Figure 3. The results showed that when the NaCas concentration was low (2∼4 wt%), the transmittance of the microspheres increased as the S/O phase content increased, and the system became more and more unstable. The better stability of the microspheres with low S/O content is due to the special structure of the free phase combined with the droplet surface. On the other hand, it was also because of the strong steric effect between the droplets and the free molecules. When the S/O phase added amount increased, the free W phase in the system gradually decreased, so the structure became unstable. With the NaCas concentration increased (6∼10 wt%), the transmittance of microspheres during centrifugation decreased significantly with the increase of the S/O phase, because there were no free W phase molecules in the aqueous phase, which reduced the occurrence of lossy flocculation.

#### Relationship between particle size, Zeta-potential, and instability index

**FIGURE 4:**
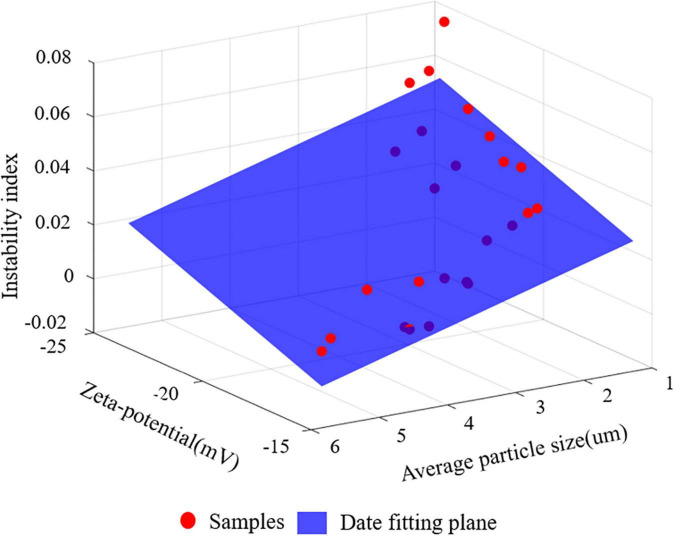
The relationship between instability index and mean particle size and Zeta-potential. The red dot represents the samples and the blue plane is the data fitting plane.

Linear regression analysis were used to fit the particle size, potential value and instability index of S/O/W emulsions stabilized by different concentrations of NaCas and different S/O phase additions, and the results were shown in Figure 4.

Three models were constructed to analyze the relationship between instability index and particle size and Zeta-potential value (Model I), the relationship between instability index and particle size (Model II), and the relationship between instability index and Zeta-potential value (Model III). Model I was modeled using two variables: instability coefficient = −0.4612 × particle size−0.4591 × potential value. In terms of the absolute value of the coefficients of the regression equation, the coefficient of 0.4612 for particle size was greater, indicating a greater correlation between particle size and instability index, but the difference between the two was smaller. The linear regression model was established with a single feature in Models II and Model III, which further showed that grain size distribution was more correlated with stability, but the difference was small. The *R*^2^ of model one (0.7262) was greater than the *R*^2^ of Models II and III (0.6481, 0.6473), making it necessary to model using both variables, and considering both features simultaneously provides greater accuracy in determining the S/O/W emulsions instability coefficient.

In summary, although particle size has a greater degree of correlation with the instability coefficient, the difference in correlation degree between particle size and the instability coefficient and potential value and the instability coefficient was relatively small.

### Viscosity analysis

**FIGURE 5:**
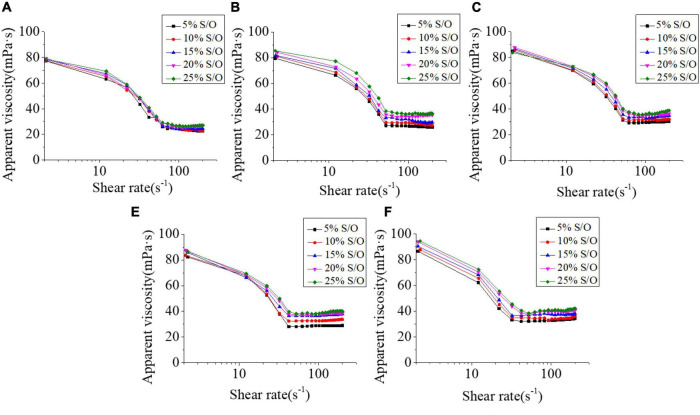
Effect of the different NaCas concentrations **(A–E)** 2 wt%, 4 wt%, 6 wt%, 8 wt%, and 10 wt%) and different S/O phase addition on the apparent viscosity of CaCO_3_ S/O/W emulsions.

The apparent viscosity of S/O/W emulsion is a significant index of the system. GEL has high viscosity at low shear rate but a sharp decrease at high shear. The pseudo-plasticity of XG is very prominent, and it is very effective in stabilizing suspension and emulsion (29). Figure 5 showed that when the S/O phase added amount was constant, the apparent viscosity increased with the NaCas concentration increased. With the NaCas concentration increased, the viscosity and stability of the S/O/W emulsions prepared by NaCas-GEL-XG ternary composite increased. Our previous studies have found that low NaCas concentration could not form a three-dimensional network structure (22), while the binary compound of NaCas-XG formed a gelatinous network structure (28). The addition of GEL could effectively improve the gel-like properties of NaCas and XG, and promoted the formation of the honeycomb three-dimensional network structure of the S/O/W emulsion, and it is a benefit to improve the viscosity and stability of the system. When NaCas concentration was constant, shear thinning occured at low S/O phase addition amount, and the system viscosity increased at high S/O phase addition amount, which was related to the fact that the O phase itself has a certain viscosity.

### Friction coefficient

Stick-slip effects are repeated sticking and sliding events which reflect intermittent static and dynamic friction phases in jagged sliding force curves and the stick-slip patterns can be influenced by contact surfaces, lubricant, and their interactions (30). The tribological analysis is a supplement to the study of rheological properties to further explain the lubrication behavior of the system. The addition of GEL and XG directly affected the particle size of droplets in S/O/W emulsions. The mixed system was also a pseudoplastic solution, which showed a downward trend with the increase in shear rate.

**FIGURE 6:**
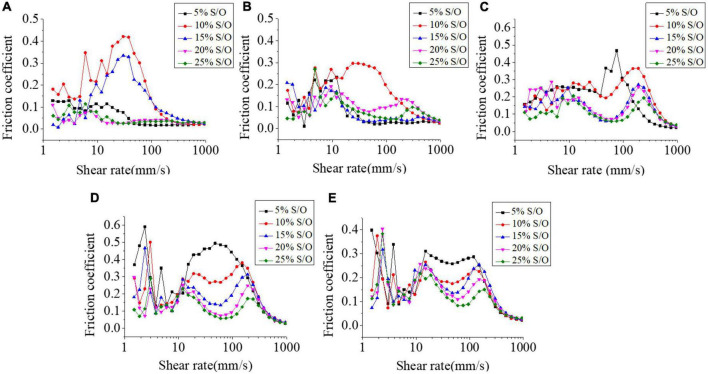
Effect of different NaCas concentrations **(A–E)** 2 wt%, 4 wt%, 6 wt%, 8 wt%, and 10 wt%) and different S/O phase addition on the friction coefficient of CaCO_3_ S/O/W emulsions.

The results of different NaCas concentrations and S/O phase addition amount on the friction coefficient on S/O/W emulsions were shown in Figure 6. The S/O/W emulsions were contacted with the contact surface firstly, the friction coefficient under low shear rate was higher, and then reduced with the shear rate increased. A high shear rate will destroy the intermolecular forces, so that more emulsion particles gather on the surface of the contact surface, forming a certain resistance, and then the viscosity behavior plays a dominant role, which reduces the friction coefficient of the whole system. When the NaCas concentration was lower, the S/O/W emulsion presented a low friction resistance. The particles in the boundary zone can effectively reduce the friction resistance of the system, and the overall trend was low. The friction coefficient was higher when NaCas concentration increased, and it was hypothesized that in the presence of GEL and XG, the emulsion particles gather inhomogeneous on the surface of the contact surface, and the overall friction coefficient became larger. With the S/O phase addition increased, the S/O/W emulsion friction coefficient decreased and there was a direct correlation between the grease content and the perceived smoothness of the sample in the mouth. When the oil content was lower, the friction coefficient of the emulsions was higher. When the oil content increased, the friction coefficient of the emulsion decreased.

### Microstructure

#### Confocal scanning laser microscopy

**FIGURE 7:**
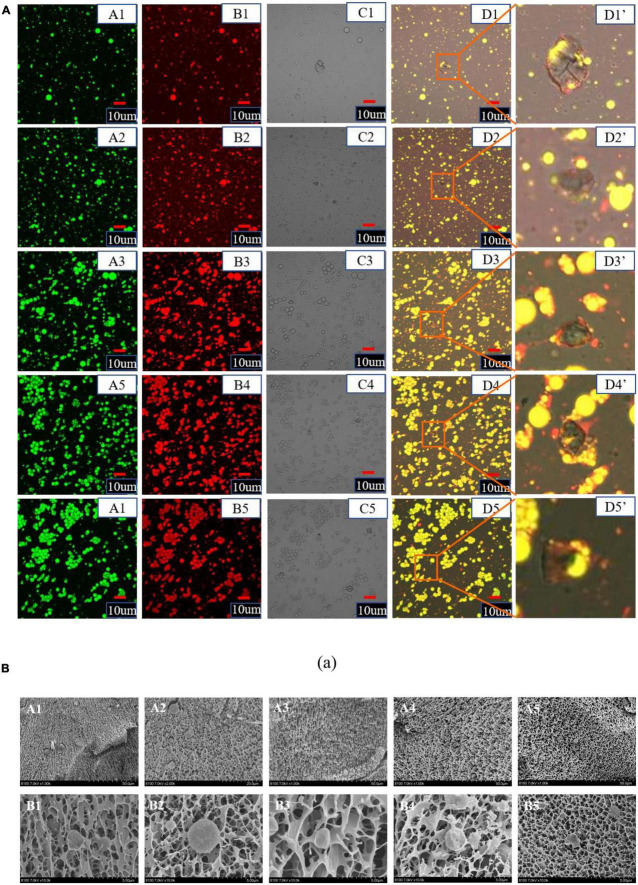
**(A)** Confocal laser scanning microscope images of the CaCO_3_ S/O/W emulsions (A, Oil phase was stained with Nile red, excitation at 488 nm; B, Protein phase was stained with Nile blue, excitation at 635 nm (ii); C, bright field view; D, combined image; D’, high magnified image). Scale bar: 10 μm. (1–5: 2, 4, 6, 8, and 10 wt% NaCas). **(B)** Cryo-SEM images of the CaCO_3_ S/O/W emulsions (A, 5000×; B, 20000×). (1–5: 2, 4, 6, 8, and 10 wt% NaCas).

Confocal scanning laser microscopy can realize imaging from macro to micro and super-resolution microscopic observation. The effect of different NaCas concentrations on the microstructure of NaCas-GEL-XG ternary composite stabilized S/O/W emulsion was observed from a relatively large field of view by CLSM, and the results were shown in Figure 7A. With the NaCas concentration increased, S/O/W emulsions have a smaller particle size and a more uniform overall distribution, which was consistent with the results in Figure 1. At low NaCas concentrations (2∼4 wt%), the distribution of S/O/W emulsions was relatively uniform and the dispersion stability was good. With the NaCas concentration increased (6∼10 wt%), the viscosity of the system increased and even agglomeration occurred, but the stability was not good. Therefore, when the concentration of NaCas was too high and the ternary combination of GEL and XG, the stability of S/O/W emulsions became worse, which was consistent with the results in Figure 3.

#### Cryo-scanning electron microscopy

Cryo-scanning electron microscopy showed the cross-sectional structure and attachment of S/O/W emulsions and observed the microstructure of S/O/W emulsions from a more microscopic perspective. The effects of different NaCas concentrations on the Cryo-SEM microstructure were shown in Figure 7B. When the NaCas concentration was low, S/O/W emulsions can form a 3D network structure. With the NaCas concentration increased, the microsphere honeycomb three-dimensional spatial network structure became more and more compact. With low NaCas concentration, the S/O/W emulsions could form a dense three-dimensional network space structure, indicating that the emulsion constructed with low NaCas concentration had good stability. In addition, low NaCas concentration could not form a three-dimensional network structure (22), while the binary compound of NaCas-XG formed a gelatinous network structure (28). The addition of GEL could effectively improve the gel-like properties of NaCas and XG, and promoted the formation of the honeycomb three-dimensional network structure of the S/O/W emulsion, and it is a benefit to improve the stability of the system.

### Stability mechanism analysis

#### X-ray diffraction analysis

**FIGURE 8:**
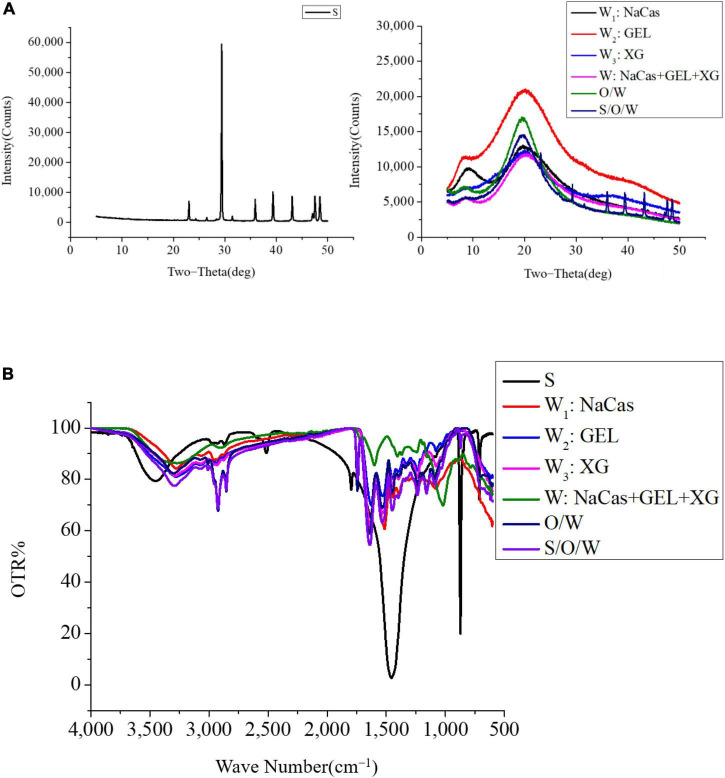
The XRD **(A)**, infrared spectral analysis **(B)** of the CaCO_3_ S/O/W emulsions.

X-ray diffraction analysis was used to characterize that the CaCO_3_ was embedded in the S/O/W emulsions, and the results were shown in Figure 8A. XRD analysis showed that the CaCO_3_ was calcite structure, and the diffraction peaks were 23, 29, 36, 39, 43, 47.5, and 48.5, respectively (31). The NaCas (W_1_ phase) diffraction peaks were 10 and 20, respectively. The GEL (W_2_ phase) crystal characteristic peaks were 7.2 and 20.2. The XG (W_3_ phase) crystal characteristic peak was 20. The diffraction peaks of NaCas-GEL-XG (W phase) were 7.2 and 20.2, which was consistent with those of GEL. The XRD results of O/W emulsions were consistent with the W phase, indicating that a better oil-in-water structure can be formed without adding calcium carbonate. The diffraction peaks of S/O/W emulsion were 7.2, 20, 23, 29, 36, 39, 43, 47.5, and 48.5, respectively, indicating that the characteristic peak of CaCO_3_ was partially covered and the intensity of the characteristic peak was weakened due to physical embedding. Compared with the S/O/W emulsions with stable W phases of NaCas, NaCas-GEL, and NaCas-XG, the S/O/W emulsions with a stable ternary mixture of NaCas-GEL-XG have enhanced the embedding effect of CaCO_3_ (28, 32).

#### Infrared spectral analysis

The position and intensity of absorption peak in the infrared spectrum reflect the characteristics of molecular structure and can be used to identify the structural composition of substances or determine their chemical groups. Figure 8B showed that the C-O asymmetric stretching and symmetric vibration peak were at 1796.99 and 1456.26 cm^–1^, the out-of-plane and in-plane bending vibration peak were at 872.54 and 712.67 cm^–1^.

The O-H and C-H bond stretching vibration of NaCas (W_1_ phase) were at 3275.78 and 2930.05 cm^–1^, the amide I band and amide II stretching vibration were at 1600–1700 and 1400–1550 cm^–1^ (27). GEL (W_2_ phase) has three distinctive absorption peaks of the amide band, which were the stretching vibration of −C = O at 1629.01 cm^–1^ (amide I band), the -N-H bending vibration at 1521.34 cm^–1^ (amide II bands), and the stretching vibration of C-H at 1235.44 cm^–1^ (amide III bands) (33). The –COO– asymmetric and symmetric stretching vibrations of XG (W_3_ phase) were at 1599.68 and 1405.46 cm^–1^, and the C-O stretching vibration was at 1019.76 cm^–1^. The -OH stretching vibration absorption peak shifted from 3275.78 to 3278.73 cm^–1^, which was indicated that there was hydrogen bond interaction between NaCas, GEL, and XG molecules (34). The N-H bending vibration of NaCas disappears at 1513.56 cm^–1^. The absorption peak of -N-H bending vibration at 1521.34 cm^–1^ disappears, and the absorption peak at 1531.54 cm^–1^ appears, which indicates that the –NH^3+^ vibration peak in the original absorption peak disappears, and the wave number is blue-shifted. This indicates the disappearance of the –NH^3+^ vibrational peak in the original absorption peak and the blue shift of the wave number; the absorption peaks of the –COO– stretching vibration of XG at 1599.68 and 1405.46 cm^–1^ also disappeared; these results confirmed the electrostatic interaction between GEL and –NH^3+^, XG, and –COO–.

#### Molecular docking analysis

**FIGURE 9:**
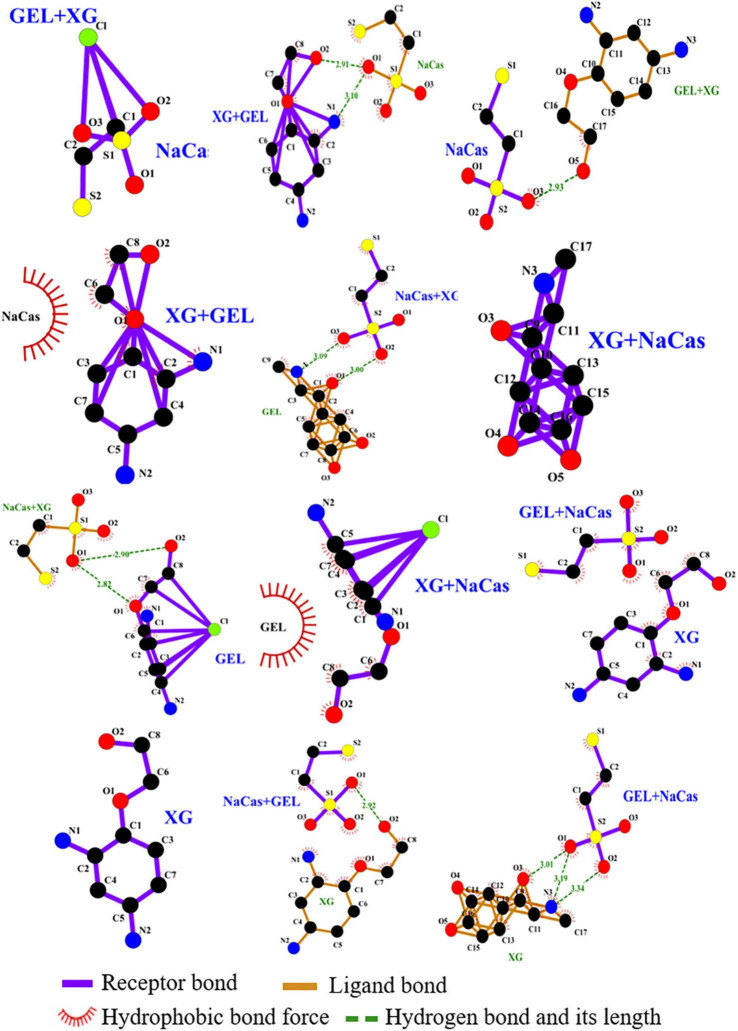
Molecular interactions among NaCas, GEL, and XG. Purple represents the receptor bond, brown represents the ligand bond, green represents hydrogen bond and its length, and red represents hydrophobic bond force.

Molecular docking technology can effectively predict the binding mode between proteins and ligands, which can be compared with infrared spectroscopy and Raman spectroscopy analysis for verification (35). Computer simulation technology was first applied in the direction of rapid discovery of chemical entities with potential interaction between drugs and target proteins, and has been applied in the field of food in recent years (35, 36). Through the principles of energy matching and spatial matching, molecular docking technology screens the conformation with the lowest energy and the most reasonable spatial matching among all docking conformations according to the inherent structural information of proteins and small molecules (37). The affinity information and docking site information of the interaction between any two compounds can be obtained by molecular docking analysis. The interaction of NaCas, GEL, and XG was visualized by molecular docking technology, and the results were shown in Figure 9.

Table 3 showed the summary of molecular interactions among NaCas, GEL and XG. When NaCas was the receptor and XG + GEL was the ligand, the binding energy was −0.2 kJ/mol, and there was an electrostatic interaction. When GEL + XG was the receptor and NaCas was the ligand, the binding energy was −2 kJ/mol, and the hydrogen bond length of O5 on GEL + XG and O3 on NaCas was 2.93 Å, indicating hydrogen bond interaction between them. When XG + GEL was the receptor and NaCas was the ligand, the binding energy was −1.4 kJ/mol, and there was a Hydrophobic interaction. The molecular docking prediction model was consistent with the results of infrared spectroscopy analysis. Hydrogen bond interaction, electrostatic interaction and hydrophobic interaction exist among NaCas, GEL and XG ternary in phase W, which may help to further improve the stability of S/O/W emulsion system.

**TABLE 3:** Summary of molecular interactions among NaCas, GEL, and XG.

System	Receptor	Ligand	Affinity (kJ/mol)	Intermolecular interaction mode
1	NaCas	GEL+XG	−0.2	Electrostatic interaction
2	NaCas	XG+GEL	−1.3	Hydrogen bond interaction
3	GEL+XG	NaCas	−2	Hydrogen bond interaction
4	XG+GEL	NaCas	−1.4	Hydrophobic interaction
5	GEL	NaCas+XG	−1.5	Hydrogen bond interaction
6	GEL	XG+NaCas	−2.4	Electrostatic interaction
7	NaCas+XG	GEL	−0.4	Hydrogen bond interaction
8	XG+NaCas	GEL	−0.4	Hydrophobic interaction
9	XG	NaCas+GEL	−1.4	Electrostatic interaction
10	XG	GEL+NaCas	−0.3	Electrostatic interaction
11	NaCas+GEL	XG	−1.3	Hydrogen bond interaction
12	GEL+NaCas	XG	−2.5	Hydrogen bond interaction

## Conclusion

Our research showed that when the NaCas concentration was 2 wt% and the S/O phase addition was 5%, the S/O/W emulsions prepared by NaCas-GEL-XG ternary composite were more stable with physical stability. The results of microstructure indicate that the CaCO_3_ powder can be effectively embedded in the S/O/W emulsion liquid system. The molecular interaction analysis and molecular docking indicated that there were electrostatic, hydrophobic interaction, and hydrogen bond interaction between W phase (NaCas, GEL, and XG), which could improve the stability of S/O/W emulsions.

## Data availability statement

The original contributions presented in this study are included in this article/supplementary material, further inquiries can be directed to the corresponding author/s.

## Author contributions

DX: writing – review and editing, methodology, and project administration. JZ: data curation, writing – original draft, investigation, validation, and formal analysis. YC: conceptualization, project administration, and supervision. All authors contributed to the article and approved the submitted version.
